# Effect of preservation solution and distension pressure on saphenous vein’s endothelium

**DOI:** 10.1093/icvts/ivac124

**Published:** 2022-05-16

**Authors:** Matheus Duarte Pimentel, José Glauco Lobo Filho, Heraldo Guedis Lobo Filho, Emílio de Castro Miguel, Sergimar Kennedy Pinheiro Paiva, João Igor Silva Matos, Matheus Augusto Mesquita Fernandes, Francisco Vagnaldo Fechine Jamacaru

**Affiliations:** 1 Department of Surgery, Federal University of Ceará, Fortaleza, Brazil; 2 Department of Metallurgical Engineering and Materials (DEMM) and Analytical Center, Federal University of Ceará, Fortaleza, Brazil; 3 Federal University of Ceará, Fortaleza, Brazil

**Keywords:** Coronary artery bypass grafting, Saphenous vein, Preservation solution, Distension pressure

## Abstract

**OBJECTIVES:**

Approaches to improve saphenous vein (SV) patency in coronary artery bypass graft (CABG) surgery remain relevant. This study aimed to evaluate the effects of different preservation solutions and different pressures of intraluminal distention on the endothelium of SV segments in CABG.

**METHODS:**

Forty-two SV segments obtained from 12 patients undergoing CABG were divided into 7 groups. Group 1 (control) was prepared without preservation or intraluminal distension, while the other 6 groups were preserved in autologous heparinized autologous arterial blood or normal saline (NS), with distention pressures 30, 100 and 300 mmHg. To assess the effects of using these solutions and pressures on the endothelium, the grafts were analysed by scanning electron microscopy, with the measurement of endothelial damage degree.

**RESULTS:**

Segments in group 1 showed minimal endothelial damage. SV grafts preserved with NS had significantly greater endothelial damage both compared to the control group and compared to groups preserved with autologous arterial blood (*P *<* *0.001). Segments distended with pressures up to 100 mmHg showed less damage when compared to those distended at 300 mmHg, with the ones subjected to higher pressures presenting a maximum degree of damage, with considerable loss and separation of endothelial cells, extensive foci of exposure of the basement membrane and numerous fractures of the intimate layer, without differences regarding the solution used.

**CONCLUSIONS:**

Preparation of SV using NS and with intraluminal distension pressures above 100 mmHg is factors related to increased damage to the venous endothelium.

**Figure:**
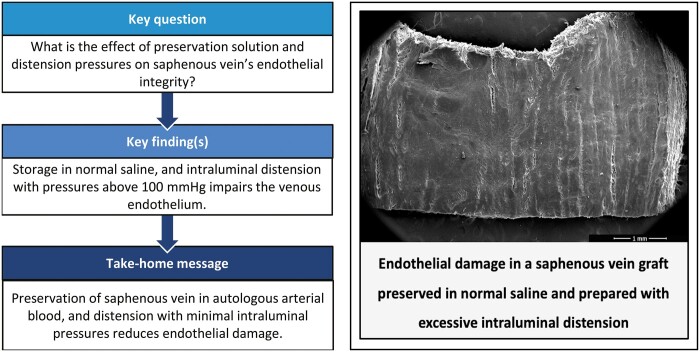

## INTRODUCTION

Coronary artery disease remains the disease with the highest mortality worldwide, with coronary artery bypass grafting (CABG) being the procedure of choice in the treatment of complex multivessel coronary artery disease [1, 2]. Despite increasing recommendations for the exclusive use of arterial grafts in CABG, the saphenous vein (SV) remains widely used [2, 3].

The development of venous graft disease is one of the main limitations related to the use of these grafts, as this pathophysiological process leads to gradual occlusion of the vascular lumen, potentially resulting in venous graft failure (VGF) [4, 5]. This event, present in up to 25% of the SV grafts in the first year, and in up to 40–50% of these grafts in 10 years, is associated with a higher incidence of unfavourable outcomes, such as death, acute myocardial infarction and need for repeated revascularization [5].

The perioperative preservation of the endothelial integrity is a relevant factor for reducing VGF rates, enhancing the importance of strategies to minimize damage to the SV [6–8]. Approaches such as the no-touch technique result in less damage to the intimal layer, lower rates of early graft occlusion and improved long-term results, sometimes even similar to those obtained with arterial grafts [6, 7].

Selection of storage solutions for graft preservation and limitation of intraluminal distension pressures are simple, cost-effective methods with experimental and clinical evidence of reducing endothelial damage to venous grafts [6, 9, 10]. However, there is no consensus regarding standardization of these aspects in the literature. In addition, studies usually assess, separately, the impact of these factors on endothelial integrity, with few reports on the joint assessment of these features [10, 11].

This study aimed to evaluate, using scanning electron microscopy (SEM), the effects of using different graft preservation solutions and distinct intraluminal distension pressures on the endothelial integrity of SV segments in CABG.

## PATIENTS AND METHODS

### Ethics statement

This study was approved by the Research Ethics Committee (Federal University of Ceará) under institutional review board number 2257035 (04 September 2017). A written informed consent form was signed by all patients.

In this prospective study, 12 non-diabetic patients, aged between 50 and 80 years, without chronic venous disease, and indication for elective CABG with the use of SV grafts, were selected from April 2019 to February 2020. Demographic data and preoperative variables are described in Table 1.

**Table 1:** Demographic data and preoperative clinical characteristics of the studied patients

Variable	*N*
Age (years) (mean ± SD)	67.67 ± 5.54
Age >75 years, *n* (%)	1 (8.3)
Male sex, *n* (%)	10 (83.4)
Previous AMI, *n* (%)	1 (8.3)
Functional class III/IV (NYHA)	0
BMI (mean ± SD)	26.83 ± 3.43
COPD, *n* (%)	1 (8.33)
Diabetes mellitus	0
Kidney failure	0
Dyslipidaemia, *n* (%)	12 (100%)
Systemic arterial hypertension, *n* (%)	11 (91.6)
Left main stem CAD, *n* (%)	2 (16.6)
Two vessel CAD, *n* (%)	2 (16.6)
Three vessel CAD, *n* (%)	8 (66.6)
LVEF ≤35%	0
Cerebrovascular disease, *n* (%)	1 (8.33)
Carotid artery disease	0
EuroScore II (mean ± SD)	1.03 ± 0.52

AMI: acute myocardial infarction; BMI: body mass index; CAD: coronary artery disease; COPD: chronic obstructive pulmonary disease; EuroScore: European System for Cardiac Operative Risk Evaluation; LVEF: left ventricle ejection fraction; NYHA: New York Heart Association; SD: standard deviation.

### Surgical procedure

The same professional harvested the SV in the lower limb through multiple open incisions, with an atraumatic technique, avoiding tension, distortion and distension of the grafts. After measuring the length of the venous segment to be used as a graft for myocardial revascularization, the remaining portion of the SV is divided into segments with ∼2 cm. The surgical team delivered this material to an examiner who prepared the graft according to each studied group requirements.

### Randomization and allocation to study groups

Considering that 7 experimental conditions were studied and 6 venous segments would be analysed in each of these conditions, one of the authors of this study produced a table with 42 allocation slots. These slots were randomized using the function RAND of Microsoft Excel, and the resulting table was used to sequentially allocate each venous segment to a study group, with their respective preparation and fixation procedures described below.

The storage solutions were used at room temperature in the operating theatre (∼20–22°C). The normal saline (NS) solution consisted of 0.9% sodium chloride.

Group 1 (control) SV segments were immediately preserved in a test tube containing a fixative solution with 4% paraformaldehyde and 2.5% glutaraldehyde and 0.1 M sodium cacodylate buffer after harvesting.

In study groups 2–7, all venous segments were distended, using a metered syringe, at a constant pressure, specific for each studied group, for 5 min, with an also specific preservation solution. Immediately after this process, the SV segments were stored in a test tube immersed in fixative solution.

SV segments from group 2 [autologous arterial blood (AAB) 30] and group 3 (NS 30) were preserved in heparinized autologous arterial blood (AAB) and NS solution, respectively, and distended following the above-described protocol, at a pressure of 30 mmHg. On the other hand, venous segments from group 4 (AAB 100) and group 5 (NS 100) were preserved in AAB and NS, respectively, and distended at a pressure of 100 mmHg. Finally, SV segments from group 6 (AAB 300) and group 7 (NS 300) were also preserved in AAB and NS solution, respectively, and distended a pressure of 300 mmHg.

### Scanning electron microscopy analysis

Venous samples stored in fixative solution were removed from their container and washed with 0.1 M sodium cacodylate buffer 3 times. Dehydration was obtained by immersion of the samples in solutions with increasing concentrations of ethanol: 50%, 70%, 90% and 100%. Once dehydrated, the samples were critical point dried (EMS 850), to remove moisture and residual solvents, and then mounted on a sample holder support for SEM (stub), using a carbon adhesive tape. Finally, the samples were sputtered coated with 20-nm gold (Quorum 150T ES).

SEM was carried out in a Quanta™ Scanning Electron Microscope 450 FEG (Thermo Fisher). The stubs containing the SV segments were placed in the microscope sample holder and then sealed. After that, a magnification of fifty times was defined and the electron beam of the microscope was turned on with a voltage of 10.0 kV for the acceleration of the electron beam, and image formation.

In each sample, a central area of 0.5 cm × 0.5 cm was selected to analyse the effects of different SV preparation techniques. From this central area, increasing magnifications: 50 times, 500 times, 1000 times and 5000 times, were adopted for a thorough evaluation, which was performed by 2 blinded observers.

The usual morphology of the SV’s endothelium and the following parameters were evaluated: separation of endothelial cells; loss of endothelial cells; basement membrane exposure; exposure of fibrillar collagen; and presence of fractures and fissures in the intimal layer.

These aspects were graded using a Likert-type scale, adapting a score proposed by Gundry *et al.* [10], objectively classifying these parameters from 0 to 4: 0 indicates the absence of morphological alterations; 1 indicates changes in up to 10% of the sample; 2 indicates changes of 10 to 25% of the sample; 3 indicates changes in 25 to 50% of the sample; and 4 indicates changes in >50% of the sample.

After analysis, the score for each of the five studied criteria was added, generating a total score for each sample, ranging from 0 to 20, with higher scores indicating greater damage to the sample studied. An average damage score for each group was also calculated.

### Data availability statement

Data from each of the venous segments were tabulated for statistical analysis (Supplementary Material). All relevant data are within the manuscript and its supporting information files.

### Statistical analysis

The measure of structural damage in SV segments, as it is a sum of scores, was considered a quantitative variable and was initially analysed using the Kolmogorov–Smirnov test to verify the normality of the distribution. As such requirement was achieved, then, for descriptive statistics, the mean and standard deviation were calculated, as well as parametric methods were used for analytical statistics. Thus, comparisons between the 7 treatment groups concerning this variable were performed using one-way analysis of variance, associated with Tukey's multiple comparisons test, to verify differences between the paired groups.

Considering that the Kolmogorov–Smirnov test has limitations when used for small samples, the adequacy of the overall structural damage variable to the normal distribution evidenced by the Kolmogorov–Smirnov test was corroborated by the analysis of the *Q*–*Q* plot, which plots the observed values of the evaluated variable versus the expected values assuming that it is a Gaussian distribution. In the case of a normal distribution, the points are expected to form a straight line approximately coincident with the line of identity. This was observed for the structural damage variable (Supplementary Material).

The 5 parameters that constitute the score for evaluating the structural damage of SV segments are ordinal variables and were expressed as median, 25th percentile and 75th percentile (interquartile range) and minimum and maximum values, with analysis by non-parametric methods. Thus, to compare the seven study groups with such variables, the Kruskal–Wallis test was used, complemented by Dunn's multiple comparisons test, to verify differences between the paired groups.

Considering the comparisons between each of the six study groups and the control group in terms of overall structural damage, we have calculated the power provided by the samples to detect the observed differences. Thus, considering the comparisons between each study group versus the control group, it is observed that the differences were significative. As the effects of interventions were expressive, inducing considerable differences, small samples were able to detect such differences.

From these analyses, it can be concluded that the sample size used provided a power >90%, at a significance level of 5%, to detect the statistically significant differences observed between the study groups and the control group in relation to the main measure of effect (structural damage score).

In all analyses, two-tailed tests were used, establishing the level of significance at 0.05 (5%), considering, therefore, a *P*-value of <0.05 as statistically significant. The software GraphPad Prism version 8.0 (GraphPad Software, SanDiego, California, USA) and IBM SPSS Statistics version 23.0 (IBM Corp., Armonk, NY, USA) were used to carry out the statistical procedures and to prepare the graphs.

## RESULTS

**Figure 1:**
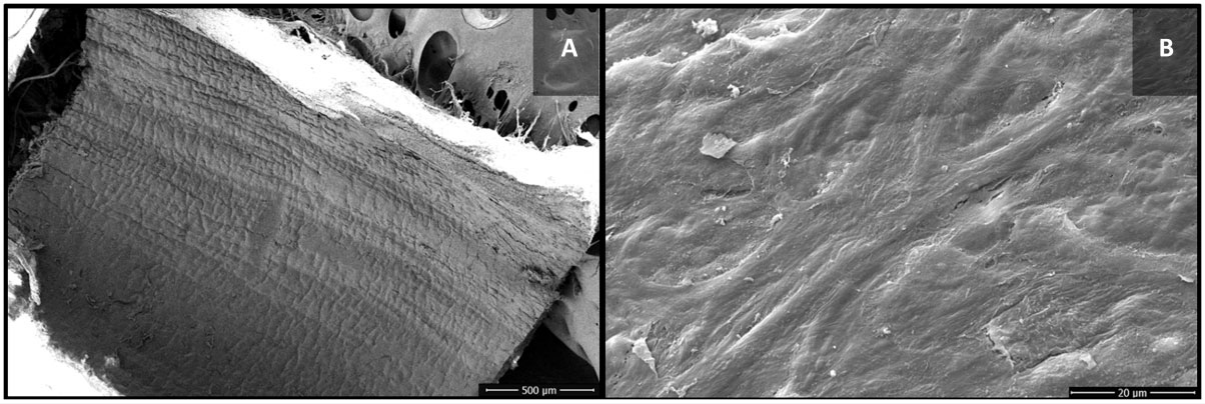
Scanning electron microscopy images of the luminal surface of the control group saphenous vein segments. (**A**) Venous endothelium with no signs of cell damage. (**B**) Intimal layer with preserved endothelial cells.

SV segments from the control group were analysed shortly after surgical excision, without the influence of graft preparation methodologies. In 4 of the 6 samples studied, complete integrity of the venous intimal layer was noted. In 2 samples, however, occasional separation of endothelial cells was observed (Fig. 1).

**Figure 2:**
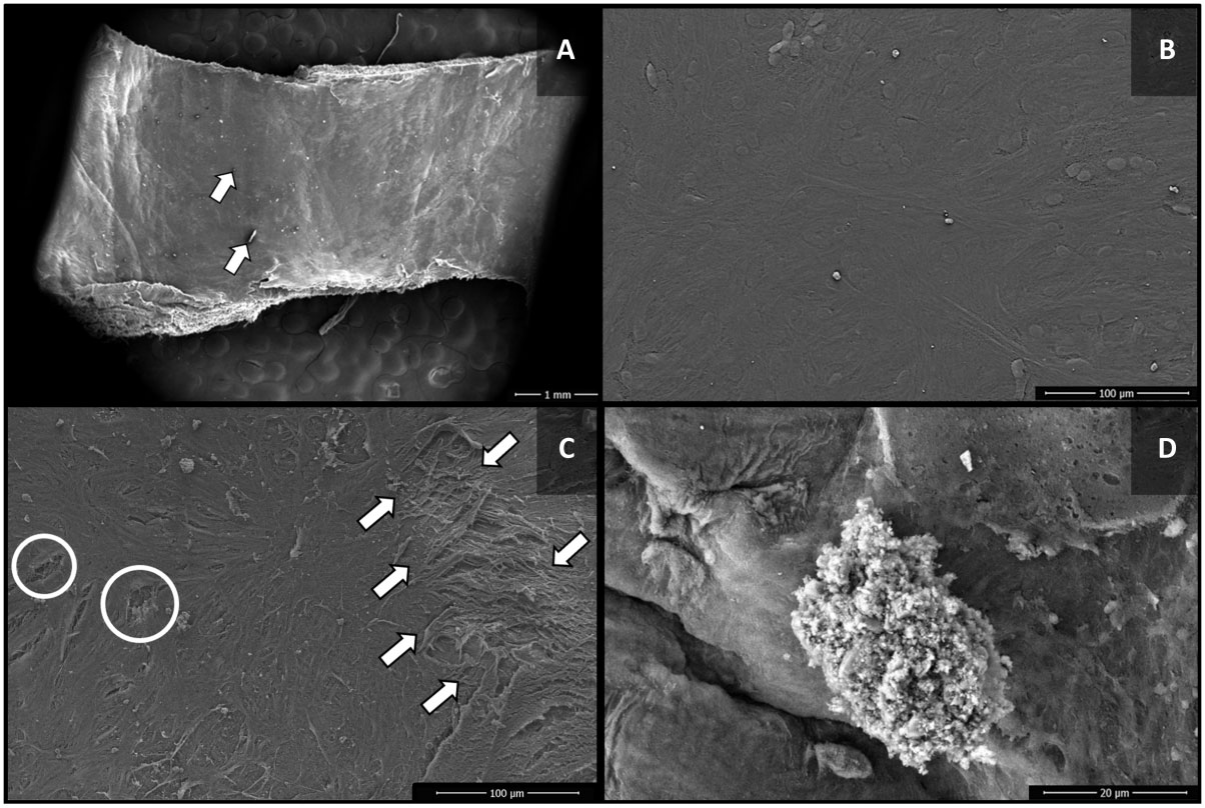
Scanning electron microscopy images of the luminal surface of saphenous vein segments of groups 2 (autologous arterial blood 30) and 3 (normal saline 30). (**A**) Group 2 (autologous arterial blood 30)—integrity of most of the endothelium, with discrete artefacts from the preparation for microscopic analysis (arrow). (**B**) Group 2 (autologous arterial blood 30)—intact endothelium. (**C**) Group 3 (normal saline 30)—marked separation of endothelial cells with exposure of subendothelial layers (arrow), in addition to fissures in the cellular intima layer (circle). (**D**) Group 3 (normal saline 30)—presence of a cluster of fibrin and platelets on the endothelial surface.

SV segments from group 2 (AAB 30) showed minor alterations in the endothelial surface, with occasional separation and/or loss of endothelial cells, and rare exposure of the basement membrane. There was no exposure of collagen fibres or fissures in the intimal layer (Fig. 2A and B). In group 3 (NS 30), however, there was an increased degree of separation and loss of endothelial cells (Fig. 2C), with the heterogeneity of cellular distribution, with endothelial loss, presence of fissures in the intimal layer and exposure of collagen fibres, with the presence of platelet clusters (Fig. 2D).

**Figure 3:**
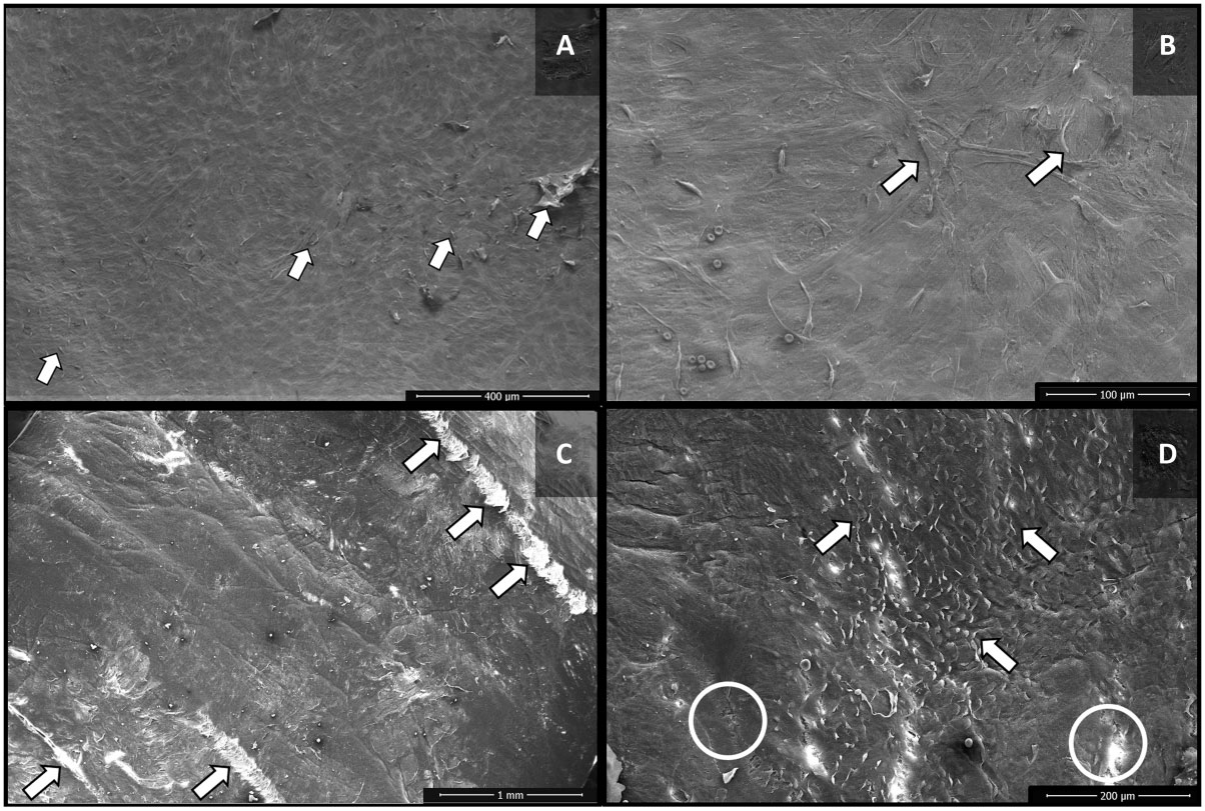
Scanning electron microscopy images of the luminal surface of saphenous vein segments of groups 4 (autologous arterial blood 100) and 5 (normal saline 100). (**A**) Group 4 (autologous arterial blood 100)—occasional separation of endothelial cells (arrow). (**B**) Group 4 (autologous arterial blood 100)—separation of endothelial cells, with exposure of the basement membrane (arrow). (**C**) Group 5 (normal saline 100)—areas with marked endothelial damage, including exposition of collagen fibres (arrows). (**D**) Group 5 (normal saline 100)—elongated pattern of the endothelial cells, with separation of these cells (arrows), in addition to fissures in the intima layer (circles).

SV segments from group 4 (AAB 100) showed marked separation of endothelial cells and exposure of the basement membrane when compared to group 2 (Fig. 3A and B). In group 5 (NS 100), differently, there was a considerable loss of endothelial cells, with frequent exposure of the collagen matrix, and fissures in the intima layer (Fig. 3C). In some venous segments, a pattern of elongation of the endothelial cells was also noted (Fig. 3D).

**Figure 4:**
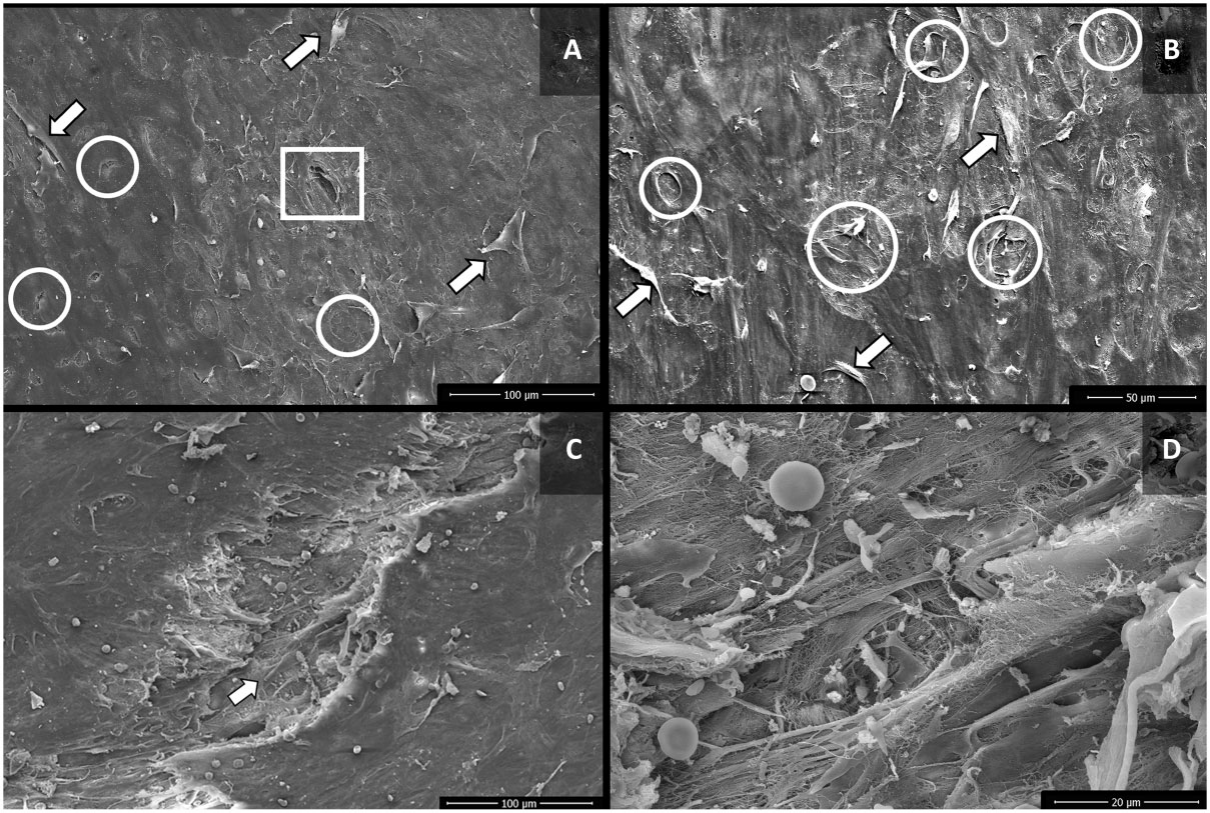
Scanning electron microscopy images of the luminal surface of saphenous vein segments of groups 6 (autologous arterial blood 300) and 7 (normal saline 300). (**A**) Group 6 (autologous arterial blood 300)—Loss and separation of endothelial cells (arrows), with exposure of the basement membrane (circles), and of collagen fibres (square). (**B**) Group 6 (autologous arterial blood 300)—separation of endothelial cells (arrows), and exposure of basement membrane (circle). (**C**) Group 7 (normal saline 300)—fissure in the intima layer (arrow), with exposure of the subendothelial layers. (**D**) Group 7 (normal saline 300)—microenvironment of fissure of the endothelial layer, with disarrangement of collagen fibres, and presence of red blood cells, fibrin and platelet agglomerates.

Venous segments from group 6 (AAB 300) presented deformation of the usual architecture of the intima layer, with extensive loss of endothelial cells, and exposure of the basement membrane, but with infrequent exposure of collagen fibres (Fig. 4A and B). In group 7 (NS 300), practically the entire length of the analysed venous segments showed a pattern of separation of endothelial cells, with subsequent exposure of subendothelial layers, presence of considerable fissures in the intima layer, with fibrin and platelet adhered to exposed collagen fibres (Fig. 4C and D).

**Figure 5:**
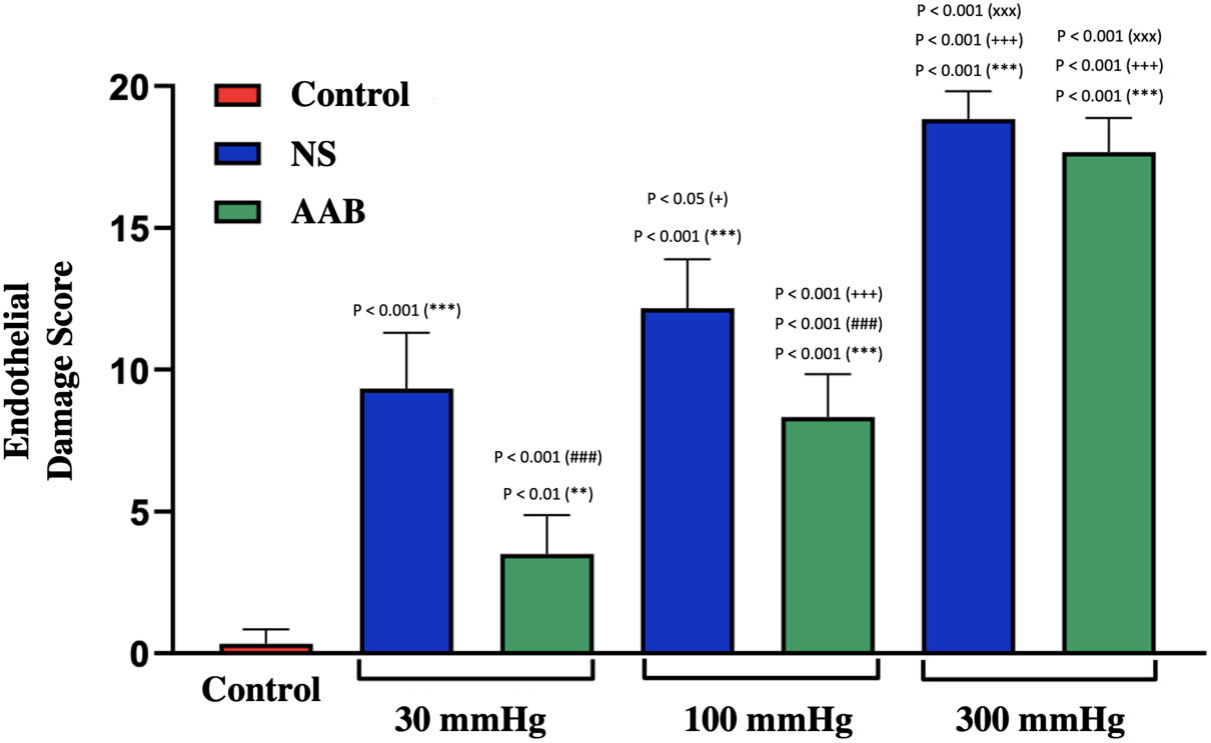
Evaluation of the degree of endothelial damage in saphenous vein segments with different storage solutions and intraluminal distension pressures. The data correspond to the mean and standard deviation of measurements performed in each group. The symbols **(*P *<* *0.01) and ***(*P *<* *0.001) denote statistically significant differences in relation to the control group, while the symbol ^###^(*P *<* *0.001) means statistically significant differences in relation to the group stored with normal saline at the same distension pressure. The symbols ^+^(*P *<* *0.05) and ^+++^(*P *<* *0.001) indicate statistically significant differences in relation to the group submitted to distension pressure of 30 mmHg in the same storage solution, while the symbol ^xxx^(*P *<* *0.001) corresponds to statistically significant differences in relation to the group submitted to distension pressure of 100 mmHg in the same storage solution.

The use of NS or AAB, as well as the distension of the SV with intraluminal pressures of 30, 100 and 300 mmHg, was related to different patterns of damage to the endothelium (Fig. 5).

It was found that, compared to NS as a storage solution, preservation with AAB significantly reduced the overall structural damage for distension pressures of 30 and 100 mmHg. Furthermore, irrespective of the storage solution, the overall structural damage increased significantly with a distension pressure of 300 mmHg.

It was also analysed how different storage solutions and increasing intraluminal distension pressures affected each of these parameters (Table 2).

**Table 2:** Detailed evaluation of the degree of endothelial damage in saphenous vein segments with different storage solutions and intraluminal distension pressures

Parameter	Control, median (IQR), *n* = 6	30 mmHg		100 mmHg		300 mmHg	
		NS, median (IQR), *n* = 6	AAB, median (IQR), *n* = 6	NS, median (IQR), *n* = 6	AAB, median (IQR), *n* = 6	NS, median (IQR), *n* = 6	AAB, median (IQR), *n* = 6
Separation of endothelial cells	0.00 (0.00–1.00)	2.00 (2.00–3.00)	2.00 (1.75–2.00)	3.00 (2.75–3.00)	2.50 (2.00–3.00)	4.00 (4.00–4.00)^a^	4.00 (4.00–4.00)^a,d^
Loss of endothelial cells	0.00 (0.00–0.00)	2.00 (1.75–3.00)	1.00 (1.00–1.00)	3.00 (3.00–3.00)^b^	2.00 (2.00–2.00)	4.00 (4.00–4.00)^a^	4.00 (4.00–4.00)^a,e^
Basement membrane exposure	0.00 (0.00–0.00)	2.00 (1.75–2.25)	0.00 (0.00–0.25)	2.50 (1.75–3.00)	2.00 (1.00–2.00)	3.50 (3.00–4.00)^b^	4.00 (4.00–4.00)^a,f^
Exposure of fibrillar collagen	0.00 (0.00–0.00)	2.00 (1.00–2.00)	0.00 (0.00–0.00)	2.50 (2.00–3.00)^c^	1.00 (0.00–1.00)	4.00 (4.00–4.00)^a^	3.00 (2.00–3.00)^c,d^
Fissures in the intimal layer	0.00 (0.00–0.00)	1.00 (1.00–1.25)	0.00 (0.00–1.25)	1.50 (1.00–2.00)	1.50 (1.00–2.00)	3.50 (2.75–4.00)^a^	3.00 (2.00–4.00)^a,d^

Superscript letters ^a^ (*P *<* *0.001), ^b^ (*P *<* *0.01) and ^c^ (*P* < 0.05) denote statistically significant differences in relation to the control group and ^d^ (*P *<* *0.05), ^e^ (*P *<* *0.05) and ^f^ (*P *<* *0.001) indicate statistically significant differences in relation to the group subjected to distension pressure of 30 mmHg and exposed to AAB (Dunn's comparison test).

AAB: autologous arterial blood; IQR: interquartile range; NS: normal saline.

## DISCUSSION

Several pathogenic factors are involved in the development of endothelial damage in the venous grafts [5, 12, 13]. Hofer *et al.* [13], in 1981, illustrated that morphological changes in SV grafts used in CABG may be due to 5 factors predominantly: changes intrinsic to the patient, which occur in the endothelium due to ageing; mechanical injury during graft excision; injuries due to hypoxia; injuries resulting from distension of the grafts; and injuries resulting from the characteristics of the SV preservation solution.

There is evidence that in the population of patients with type 2 diabetes mellitus, there is a degree of preoperative endothelial damage, with intimal hyperplasia and endothelial degeneration, which was significantly more prevalent [14]. Considering that, diabetic patients were not included in this study, to reduce biases arising from possible pre-existing endothelial damage.

Since the beginning of the systematic use of SV in CABG, several authors have sought to develop techniques that could reduce the endothelial damage to the SV. These were mainly focused on three aspects: reduction of direct trauma during harvesting; adoption of more physiological storage solutions; and use of lower distension pressures [12, 15].

Selection of the ideal graft preservation solution stills a debateable subject in CABG, and even though evidence about the potentially harmful effects of the use of NS is vast, this solution is still used for the storage of vascular grafts, due to its availability and ease of use, and the absence of consensus and studies that effectively indicate the superiority of other storage solution [5, 6, 16, 17].

Buffered saline solutions, organ preservation solutions, cardioplegic solutions and specific graft preservation solutions are subject of studies regarding their impact on venous endothelium integrity [17–20]. Harskamp *et al.* [11], in 2014, illustrated reduction in VGF and improved clinical results when buffered saline solutions were used, when compared to SV preservation with NS or autologous blood. Furthermore, Perrault *et al.* [18] reported reduction in wall thickness and in VGF-related in SV grafts prepared with DuraGraft, a balanced saline solution.

Our study illustrated that SV segments preserved in a normothermic saline solution presented considerable endothelial damage when compared to those preserved with AAB [10, 13]. This damage to the endothelial morphology also results in the impairment of cellular functionality, with deterioration in contractile capacity, and in endothelium-dependent vasodilation [19]. There are also indications that the preservation of the SV with AAB would make this graft remain in a cellular microenvironment with less expression of pro-inflammatory factors and less amount of reactive oxygen species than when preserved in NS [20].

The mechanism by which NS induces endothelial damage in vascular grafts is still discussed, but the described pathophysiological processes involve activation of a pro-inflammatory cell pathway, namely the P2X7R/p38MAPK/MK2 pathway, resulting in the loss of endothelial cell integrity and dysfunction of these cells. In addition, the fact that this unbuffered solution has an acidic pH (∼5.0) would also be a directly harmful factor to the endothelium, resulting in the loss of endothelium-dependent vascular relaxation, with lower expression of nitric oxide-producing enzymes [6, 7, 21].

The lack of consensus in the literature as to which is the superior preservation solution causes heterogeneity in the preparation of SV, which may partially explain the unsatisfactory long-term results obtained with venous grafts. To illustrate that research carried out with surgeons from 90 US hospitals showed that 28.9% of the centres used NS, 40% buffered saline solutions and 26.7% adopted AAB as a routine solution. In 88.9% of cases, the preservation solution was heparinized, and in 74.4% of cases, it remained at room temperature [22].

The intraluminal distension of the SV is the other relevant factor for its endothelial integrity. The hydrostatic pressures to which the SV is usually subjected range from 5 to 35 mmHg and may reach 50 mmHg in extreme situations. A sudden distension of this vessel at high pressures causes considerable impact in the intimal and medial layers. For example, an increase in intraluminal pressure from 10 to 100 mmHg causes a fifteen-fold increase in the circumferential stress measured on the vascular wall, which increases exponentially with higher distension pressures [6, 9, 23–25].

The effects of exaggerated luminal distension are not restricted to the endothelium. When stretched, the smooth muscle cells of the media layer of these vessels reduce their amount of adenosine triphosphate, which would be indicative of metabolic damage to these cells [6, 9]. In addition, there is a recruitment of pro-inflammatory and pro-thrombotic molecules into the vascular lumen, contributing to early thrombus formation and possible graft occlusion [24–26].

These factors illustrate the relevance of controlling the luminal distension pressure of the SV during CABG. Manual distensions, even when performed cautiously, can result in pressures of up to 400 mmHg, resulting in considerable endothelial damage to the grafts, also reducing their elasticity and capacity for tissue regeneration [21, 25]. The use of metered systems to seek distension of up to 100 mmHg would probably a more recommendable approach [5, 6].

### Limitations

This study has several limitations. Initially, this is a single-centre study, with a small sample of patients. It is also noteworthy that only the morphological aspect of the endothelial layer of the SV was evaluated when it is exposed to different preservation solutions and distending pressures, and data on cell functionality and expression of markers could not be evaluated with SEM. Furthermore, the time of exposure of the venous graft to the respective distension pressures of each group, 20 min, does not reflect what happens in surgical practice, since the venous segments are exposed for considerably shorter times; however, this time was necessary for tissue fixation to occur, allowing a reliable analysis by SEM. In addition, the structural damage score, the instrument used to evaluate each SV segment, is an effort to quantify patterns of endothelial damage that are impractical to accurately measure, and the relation between this score and unfavourable clinical outcomes can be inferred, as long-term clinical outcomes and follow-up studies evaluating VGF are not available in our study.

## CONCLUSION

In brief, structural damage to the endothelium of SV segments increases with higher distension pressure regardless of the preservation solution. Furthermore, the use of NS results in greater structural damage compared to preservation with AAB for pressures up to 100 mmHg.

## SUPPLEMENTARY MATERIAL

Supplementary material is available at *ICVTS* online.

**Conflict of interest:** none declared.

## Author contributions

**Matheus Duarte Pimentel:** Conceptualization; Data curation; Formal analysis; Investigation; Methodology; Project administration; Resources; Visualization; Writing—original draft; Writing—review & editing. **José Glauco Lobo Filho:** Conceptualization; Data curation; Formal analysis; Methodology; Supervision; Validation; Visualization; Writing—review & editing. **Heraldo Guedis Lobo Filho:** Conceptualization; Data curation; Formal analysis; Methodology; Supervision; Validation; Visualization; Writing—review & editing. Emílio de Castro Miguel**:** Conceptualization; Data curation; Formal analysis; Investigation; Methodology; Project administration; Resources; Software; Supervision; Validation; Writing—review & editing. **Sergimar Kennedy Pinheiro Paiva:** Data curation; Formal analysis; Investigation; Methodology; Project administration; Visualization; Writing—review & editing. **João Igor Silva Matos:** Investigation; Methodology; Resources; Visualization; Writing—original draft. **Matheus Augusto Mesquita Fernandes:** Investigation; Methodology; Resources; Visualization; Writing—original draft. **Francisco Vagnaldo Fechine Jamacaru:** Data curation; Formal analysis; Methodology; Writing—review & editing.

## Reviewer information

Interactive CardioVascular and Thoracic Surgery thanks Dimitrios Dougenis, Dominique Shum-Tim, Jose Lopez-Menendez and the other, anonymous reviewer(s) for their contribution to the peer review process of this article.

## Supplementary Material

ivac124_Supplementary_Material

## ABBREVIATIONS

AAB: Autologous arterial blood
AMI: Acute myocardial infarction
CABG: Coronary artery bypass grafting
NS: Normal saline
SV: Saphenous vein
SEM: Scanning electron microscopy
VGF: Venous graft failure

## References

[1] Mack M , Gopal A. Epidemiology, traditional and novel risk factors in coronary artery disease. Heart Fail Clin 2016;12:1–10. 26567971 10.1016/j.hfc.2015.08.002

[2] Neumann F , Sousa-Uva M, Ahlsson A, Alfonso F, Banning AP, Benedetto U et al; ESC Scientific Document Group. 2018 ESC/EACTS Guidelines on myocardial revascularization. Eur Heart J 2019;40:87–165.30165437

[3] D'Agostino RS , Jacobs JP, Badhwar V, Fernandez FG, Paone G, Wormuth DW et al The Society of Thoracic Surgeons adult cardiac surgery database: 2018 update on outcomes and quality. Ann Thorac Surg 2018;105:15–23. 29233331 10.1016/j.athoracsur.2017.10.035

[4] Ward AO , Caputo M, Angelini GD, George SJ, Zakkar M. Activation and inflammation of the venous endothelium in vein graft disease. Atherosclerosis 2017;265:266–74. 28865843 10.1016/j.atherosclerosis.2017.08.023

[5] de Vries MR , Simons KH, Jukema JW, Braun J, Quax PHA. Vein graft failure: from pathophysiology to clinical outcomes. Nat Rev Cardiol 2016;13:451–70. 27194091 10.1038/nrcardio.2016.76

[6] Caliskan E , de Souza DR, Böning A, Liakopoulos OJ, Choi Y, Pepper J et al Saphenous vein grafts in contemporary coronary artery bypass graft surgery. Nat Rev Cardiol 2020;17:155–69. 31455868 10.1038/s41569-019-0249-3

[7] Cheung-Flynn J , Song J, Voskresensky I, Wise ES, Liu Y, Xiong Y et al Limiting injury during saphenous vein graft preparation for coronary arterial bypass prevents metabolic decompensation. Sci Rep 2017;7:14179. 29079734 10.1038/s41598-017-13819-w PMC5660200

[8] Samano N , Dashwood M, Souza D. No-touch vein grafts and the destiny of venous revascularization in coronary artery bypass grafting—a 25th anniversary perspective. Ann Cardiothorac Surg 2018;7:681–5. 30505753 10.21037/acs.2018.05.15 PMC6219949

[9] Gooch KJ , Firstenberg MS, Shrefler BS, Scandling BW. Biomechanics and mechanobiology of saphenous vein grafts. J Biomech Eng 2018;140:020804. 10.1115/1.4038705 29222565

[10] Gundry SR , Jones M, Ishihara T, Ferrans VJ. Intraoperative trauma to human saphenous veins: scanning electron microscopic comparison of preparation techniques. Ann Thorac Surg 1980;30:40–7. 6772117 10.1016/s0003-4975(10)61200-3

[11] Harskamp RE , Alexander JH, Schulte PJ, Brophy CM, Mack MJ, Peterson ED et al Vein graft preservation solutions, patency, and outcomes after coronary artery bypass graft surgery. JAMA Surg 2014;149:798–805. 25073921 10.1001/jamasurg.2014.87 PMC4332522

[12] Bonchek LI. Prevention of endothelial damage during preparation of saphenous veins for bypass grafting. J Thorac Cardiovasc Surg 1980;79:911–5. 6768934

[13] Hofer H , Mihatsch M, Guggenheim R, Amsler B, Hasse J, Graedel E. Morphologic studies in saphenous vein grafts for aorto-coronary bypass surgery. Part I: morphology of the graft using ordinary surgical preparation techniques. Thorac Cardiovasc Surg 1981;29:32–7. 6164115 10.1055/s-2007-1023437

[14] Panetta TF , Marin ML, Veith FJ, Goldsmith J, Gordon RE, Jones AM et al Unsuspected preexisting saphenous vein disease: an unrecognized cause of vein bypass failure. J Vasc Surg 1992;15:102–12. 1728668

[15] Angelini GD , Newby AC. The future of saphenous vein as a coronary artery bypass conduit. Eur Heart J 1989;10:273–80. 2565234 10.1093/oxfordjournals.eurheartj.a059476

[16] Woodward LC , Antoniades C, Taggart DP. Intraoperative vein graft preservation: what is the solution? Ann Thorac Surg 2016;102:1736–46. 27624295 10.1016/j.athoracsur.2016.05.097

[17] Tsakok M , Montgomery-Taylor S, Tsakok T. Storage of saphenous vein grafts prior to coronary artery bypass grafting: is autologous whole blood more effective than saline in preserving graft function? Interact CardioVasc Thorac Surg 2012;15:720–5. 22753436 10.1093/icvts/ivs275 PMC3445367

[18] Perrault LP , Carrier M, Voisine P, Olsen PS, Noiseux N, Jeanmart H et al Sequential multidetector computed tomography assessments after venous graft treatment solution in coronary artery bypass grafting. J Thorac Cardiovasc Surg 2021;161:96–106. 10.1016/j.jtcvs.2019.10.115 31866081

[19] Wilbring M , Ebner A, Schoenemann K, Knaut M, Tugtekin SM, Zatschler B et al Heparinized blood better preserves cellular energy charge and vascular functions of intraoperatively stored saphenous vein grafts in comparison to isotonic sodium-chloride-solution. Clin Hemorheol Microcirc 2013;55:445–55. 24113503 10.3233/CH-131781

[20] Chen S , Chu Y, Wu VC, Tsai F, Nan Y, Lee H et al Microenvironment of saphenous vein graft preservation prior to coronary artery bypass grafting. Interact CardioVasc Thorac Surg 2019;28:71–8. 29986023 10.1093/icvts/ivy201

[21] Wise ES , Hocking KM, Eagle S, Absi T, Komalavilas P, Cheung-Flynn J et al Preservation solution impacts physiologic function and cellular viability of human saphenous vein graft. Surgery 2015;158:537–46. 26003912 10.1016/j.surg.2015.03.036 PMC4492846

[22] Williams JB , Harskamp RE, Bose S, Lawson JH, Alexander JH, Smith PK et al The preservation and handling of vein grafts in current surgical practice. JAMA Surg 2015;150:681–3. 25970819 10.1001/jamasurg.2015.0404

[23] Gusic RJ , Myung R, Petko M, Gaynor JW, Gooch KJ. Shear stress and pressure modulate saphenous vein remodeling *ex vivo*. J Biomech 2005;38:1760–9. 16023463 10.1016/j.jbiomech.2004.10.030

[24] Stigler R , Steger C, Schachner T, Holfeld J, Edlinger M, Grimm M et al The impact of distension pressure on acute endothelial cell loss and neointimal proliferation in saphenous vein grafts. Eur J Cardiothorac Surg 2012;42:e74–e79. 22906599 10.1093/ejcts/ezs402

[25] Tineli RA , Viaro F, Dalio MB, Reis GS, Basseto S, Vicente W. V D A et al Mechanical forces and human saphenous veins: coronary artery bypass graft implications. Rev Bras Cir Cardiovasc 2007;22:87–95. 17992309 10.1590/s0102-76382007000100016

[26] Chester AH , Buttery LD, Borland JA, Springall DR, Rothery S, Severs NJ et al Structural, biochemical and functional effects of distending pressure in the human saphenous vein: implications for bypass grafting. Coron Artery Dis 1998;9:143–51. 9647416

