# Supplementary material for: Effect of preservation solution and distension pressure on saphenous vein’s endothelium
Source: Interact Cardiovasc Thorac Surg. 2022 May 16;35(3):ivac124. doi: 10.1093/icvts/ivac124 (PMC9419693; doi:10.1093/icvts/ivac124)

**Supplementary Material**

**Supplementary Table 1. Damage score data from each saphenous vein segment**

| VENOUS SEGMENTS | DAMAGE SCORE |
| --- | --- |
| Segment no. 1 (Group 2) | 3 |
| Segment no. 2 (Group 3) | 12 |
| Segment no. 3 (Group 6) | 17 |
| Segment no. 4 (Group 2) | 2 |
| Segment no. 5 (Group 3) | 10 |
| Segment no. 6 (Group 1) | 1 |
| Segment no. 7 (Group 6) | 19 |
| Segment no. 8 (Group 7) | 18 |
| Segment no. 9 (Group 5) | 12 |
| Segment no. 10 (Group 4) | 9 |
| Segment no. 11 (Group 5) | 9 |
| Segment no. 12 (Group 7) | 20 |
| Segment no. 13 (Group 5) | 12 |
| Segment no. 14 (Group 4) | 7 |
| Segment no. 15 (Group 5) | 13 |
| Segment no. 16 (Group 3) | 6 |
| Segment no. 17 (Group 6) | 17 |
| Segment no. 18 (Group 5) | 13 |
| Segment no. 19 (Group 4) | 9 |
| Segment no. 20 (Group 1) | 0 |
| Segment no. 21 (Group 7) | 18 |
| Segment no. 22 (Group 6) | 16 |
| Segment no. 23 (Group 6) | 19 |
| Segment no. 24 (Group 2) | 6 |
| Segment no. 25 (Group 1) | 1 |
| Segment no. 26 (Group 1) | 2 |
| Segment no. 27 (Group 4) | 10 |
| Segment no. 28 (Group 3) | 9 |
| Segment no. 29 (Group 4) | 6 |
| Segment no. 30 (Group 1) | 0 |
| Segment no. 31 (Group 5) | 14 |
| Segment no. 32 (Group 2) | 3 |
| Segment no. 33 (Group 2) | 3 |
| Segment no. 34 (Group 7) | 18 |
| Segment no. 35 (Group 3) | 12 |
| Segment no. 36 (Group 2) | 4 |
| Segment no. 37 (Group 6) | 18 |
| Segment no. 38 (Group 3) | 10 |
| Segment no. 39 (Group 4) | 9 |
| Segment no. 40 (Group 7) | 19 |
| Segment no. 41(Group 1) | 0 |
| Segment no. 42 (Group 7) | 20 |

Data expressed as an absolute number indicating the degree of endothelial damage as follows: 0 indicates absence of morphological alterations; 1 indicates changes in up to 10% of the sample; 2 indicates changes of 10 to 25% of the sample; 3 indicates changes in 25 to 50% of the sample, and 4 indicates changes in more than 50% of the sample.

**Supplementary Table 2. Score data for each parameter of endothelial damage in each group**

| GROUP 1 - CONTROL | | | | | | |
| --- | --- | --- | --- | --- | --- | --- |
| Parameter of endotelial damage | **Damage Escore** | | | | | |
|  | SV no. 6 | SV no. 25 | SV no. 26 | SV no. 41 | SV no. 20 | SV no. 30 |
| Separation of endothelial cells | 1 | 1 | 0 | 0 | 0 | 0 |
| Loss of endothelial cells | 0 | 0 | 0 | 0 | 0 | 0 |
| Basement membrane exposure | 0 | 0 | 0 | 0 | 0 | 0 |
| Exposure of fibrillar collagen | 0 | 0 | 0 | 0 | 0 | 0 |
| Fractures and fissures in the intimal layer | 0 | 0 | 0 | 0 | 0 | 0 |
| Total Score | 1 | 1 | 0 | 0 | 0 | 0 |
| Group Damage Score | 0.33 ± 0.51 | | | | | |
|  | | | | | | |
| GROUP 2 – AAB 30 | | | | | | |
| Parameter of endotelial damage | **Damage Escore** | | | | | |
|  | SV no. 32 | SV no. 33 | SV no. 36 | SV no. 30 | SV no. 1 | SV no. 4 |
| Separation of endothelial cells | 2 | 2 | 2 | 2 | 2 | 1 |
| Loss of endothelial cells | 1 | 1 | 1 | 1 | 1 | 1 |
| Basement membrane exposure | 0 | 0 | 0 | 1 | 0 | 0 |
| Exposure of fibrillar collagen | 0 | 0 | 0 | 0 | 0 | 0 |
| Fractures and fissures in the intimal layer | 0 | 0 | 1 | 2 | 0 | 0 |
| Total Score | 3 | 3 | 4 | 6 | 3 | 2 |
| Group Damage Score | 3.5 ± 1.37 | | | | | |
|  | | | | | | |
| GROUP 3 – NS 30 | | | | | | |
| Parameter of endotelial damage | **Damage Escore** | | | | | |
|  | SV no. 2 | SV no. 5 | SV no. 28 | SV no. 35 | SV no. 16 | SV no. 38 |
| Separation of endothelial cells | 3 | 3 | 2 | 2 | 2 | 2 |
| Loss of endothelial cells | 3 | 2 | 2 | 2 | 1 | 3 |
| Basement membrane exposure | 3 | 2 | 2 | 2 | 1 | 2 |
| Exposure of fibrillar collagen | 2 | 2 | 2 | 1 | 1 | 2 |
| Fractures and fissures in the intimal layer | 1 | 1 | 1 | 2 | 1 | 1 |
| Total Score | 12 | 10 | 9 | 9 | 6 | 10 |
| Group Damage Score | 9.33 ± 1.96 | | | | | |
|  | | | | | | |
| GROUP 4 – AAB 100 | | | | | | |
| Parameter of endotelial damage | **Damage Escore** | | | | | |
|  | SV no. 10 | SV no. 29 | SV no. 27 | SV no. 14 | SV no. 19 | SV no. 39 |
| Separation of endothelial cells | 3 | 3 | 2 | 2 | 3 | 2 |
| Loss of endothelial cells | 2 | 2 | 2 | 2 | 2 | 2 |
| Basement membrane exposure | 2 | 2 | 1 | 1 | 2 | 2 |
| Exposure of fibrillar collagen | 1 | 1 | 0 | 0 | 1 | 1 |
| Fractures and fissures in the intimal layer | 2 | 1 | 1 | 2 | 1 | 2 |
| Total Score | 10 | 9 | 6 | 7 | 9 | 9 |
| Group Damage Score | 8.33 ± 1.50 | | | | | |
|  | | | | | | |
| GROUP 5 – NS 100 | | | | | | |
| Parameter of endotelial damage | **Damage Escore** | | | | | |
|  | SV no. 9 | SV no. 15 | SV no. 18 | SV no. 31 | SV no. 11 | SV no. 13 |
| Separation of endothelial cells | 3 | 3 | 3 | 3 | 2 | 3 |
| Loss of endothelial cells | 3 | 3 | 3 | 3 | 3 | 3 |
| Basement membrane exposure | 3 | 3 | 3 | 2 | 1 | 2 |
| Exposure of fibrillar collagen | 2 | 2 | 3 | 3 | 2 | 3 |
| Fractures and fissures in the intimal layer | 1 | 2 | 2 | 2 | 1 | 1 |
| Total Score | 12 | 13 | 14 | 13 | 9 | 12 |
| Group Damage Score | 12.16 ± 1.72 | | | | | |
|  | | | | | | |
| GROUP 6 – AAB 300 | | | | | | |
| Parameter of endotelial damage | **Damage Escore** | | | | | |
|  | SV no. 7 | SV no. 17 | SV no. 3 | SV no. 37 | SV no. 22 | SV no. 23 |
| Separation of endothelial cells | 4 | 4 | 4 | 4 | 4 | 4 |
| Loss of endothelial cells | 4 | 4 | 4 | 4 | 4 | 4 |
| Basement membrane exposure | 4 | 4 | 4 | 4 | 4 | 2 |
| Exposure of fibrillar collagen | 3 | 3 | 2 | 3 | 3 | 2 |
| Fractures and fissures in the intimal layer | 4 | 2 | 3 | 3 | 4 | 16 |
| Total Score | 19 | 17 | 17 | 18 | 19 | 4 |
| Group Damage Score | 17.66 ± 1.21 | | | | | |
|  | | | | | | |
| GROUP 7 – NS 300 | | | | | | |
| Parameter of endotelial damage | **Damage Escore** | | | | | |
|  | SV no. 21 | SV no. 12 | SV no. 34 | SV no. 8 | SV no. 40 | SV no. 42 |
| Separation of endothelial cells | 4 | 4 | 4 | 4 | 4 | 4 |
| Loss of endothelial cells | 4 | 4 | 4 | 4 | 4 | 4 |
| Basement membrane exposure | 4 | 4 | 3 | 3 | 3 | 4 |
| Exposure of fibrillar collagen | 4 | 4 | 4 | 4 | 4 | 4 |
| Fractures and fissures in the intimal layer | 2 | 4 | 3 | 3 | 4 | 4 |
| Total Score | 18 | 20 | 18 | 18 | 19 | 20 |
| Group Damage Score | 18.83 ± 0.98 | | | | | |

Data expressed as an absolute number indicating the degree of endothelial damage as follows: 0 indicates absence of morphological alterations; 1 indicates changes in up to 10% of the sample; 2 indicates changes of 10 to 25% of the sample; 3 indicates changes in 25 to 50% of the sample, and 4 indicates changes in more than 50% of the sample. Group damage score expressed as mean ± standard deviation.

SV: Saphenous vein segment. AAB 30: Saphenous vein preserved in autologous arterial blood distended at 30 mmHg; NS 30: Saphenous vein preserved in normal saline distended at 30 mmHg; AAB 100: Saphenous vein preserved in autologous arterial blood distended at 100 mmHg; NS 100: Saphenous vein preserved in normal saline distended at 100 mmHg; AAB 300: Saphenous vein preserved in autologous arterial blood distended at 300 mmHg; NS 300: Saphenous vein preserved in normal saline distended at 300 mmHg;

**Supplementary Graph 1. Score data for each parameter of endothelial damage in each group**

The adequacy of the overall structural damage variable to the normal distribution evidenced by the Kolmogorov-Smirnov test was corroborated by the analysis of the Q-Q plot, which plots the observed values of the evaluated variable versus the expected values assuming that it is a Gaussian distribution. In the case of a normal distribution, the points are expected to form a straight line approximately coincident with the line of identity. This fact was observed for the global structural damage variable, as shown in the graph below.


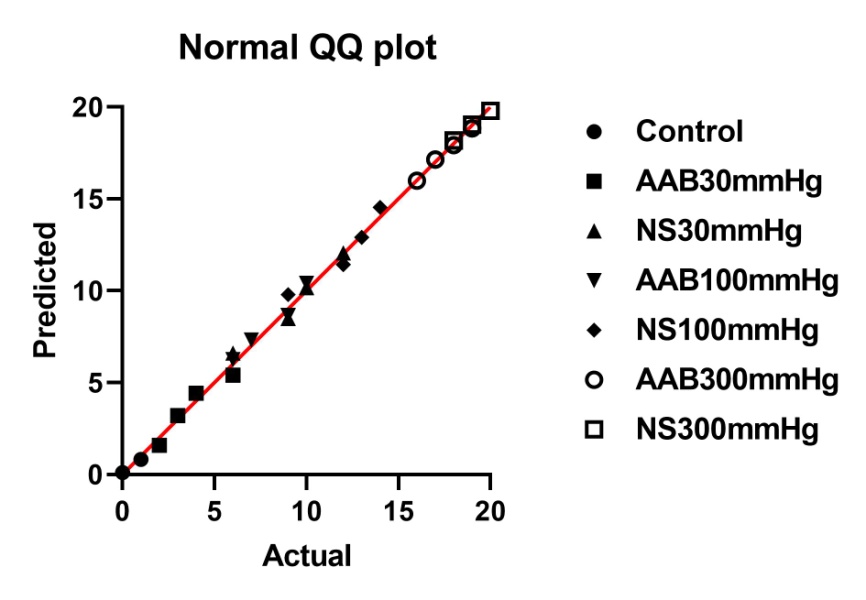

Supplement: ivac124_Supplementary_Material [file ivac124_supplementary_material.docx]
